# One-year healthcare costs after robotic-assisted and laparoscopic partial and radical nephrectomy: a cohort study

**DOI:** 10.1186/s12913-023-10111-8

**Published:** 2023-10-14

**Authors:** Kennedy E. Okhawere, Gediwon Milky, Shirin Razdan, I-Fan Shih, Yanli Li, Laura Zuluaga, Ketan K. Badani

**Affiliations:** 1 Department of Urology, Icahn School of Medicine at Mount Sinai, 1425 Madison Avenue, 6Th Floor, New York City, NY 10029 USA; 2 Intuitive Surgical, Inc, Sunnyvale, CA USA

**Keywords:** Kidney cancer, Nephrectomy, Robotic, Laparoscopic, Expenditures, Costs

## Abstract

**Objective:**

Despite the wide-spread adoption of robotic-assisted surgery (RAS), the cost–benefit implications for partial (PN) and radical nephrectomy (RN) versus laparoscopic surgery (Lap) is not well established. We sought to examine the trend of adoption and 1-year healthcare expenditure of PN and RN, and compare 1-year expenditures of RAS versus Lap for PN and RN.

**Patients and methods:**

This cohort study used the Merative^TM^ MarketScan® Databases between 2013 and 2020. A total of 5,353 patients with kidney cancer undergoing PN (2,980, 55.7%) or RN (2,373, 44.3%). We compared open-conversion, length of stay (LOS), index expenditure, 1-year healthcare expenditure and utilization, and missed work-days between RAS and Lap for PN and RN.

**Results:**

Adoption of PN increased overtime (47.0% to 55.8%), mainly driven by robotic PN increase. Among PN, RAS had lower open-conversion, shorter LOS and lower index expenditure than Lap. Among RN, RAS had shorter LOS, and similar open-conversion and index expenditures. During 1-year post-discharge, RAS had lower hospital outpatient visits (IRR = 0.92, 95% CI = 0.85, 0.99, *p* = 0.029) and office-based visits (IRR = 0.91, 95% CI = 0.86, 0.96, *p* = 0.002) for PN, translating to a 1-day less (95% CI = 0.25, 1.75, *p* = 0.008) missed from work for RAS. Following RN, RAS had lower 1-year readmission than Lap (O.R = 0.72, 95% CI = 0.55, 0.94, *p* = 0.018). RAS and Lap had comparable 1-year post-discharge expenditures for both PN (mean difference, MD = -$475, 95% CI = -$4362, $3412, *p* = 0.810) and RN (MD = -$4,204, 95% CI = -$13,837, $5430, *p* = 0.404).

**Conclusion:**

At index surgery, RAS was associated with shorter LOS for both PN and RN, and lower open-conversion and expenditures for PN. RAS and Lap had comparable 1-year total expenditures, despite lower healthcare visits for RAS.

**Supplementary Information:**

The online version contains supplementary material available at 10.1186/s12913-023-10111-8.

## Introduction

The evolution and adoption of robotic surgery have been rapid, partly owing to its clinical benefit – especially for nephron-sparing surgery in patients with localized renal cancers [1–3]. However, the discordance with the cost of utilization is arguably prohibitive, encouraging a discussion regarding its cost benefits and utility. In a previous study [4], the comparative long-term cost benefits of minimally invasive surgery, including laparoscopic approach, versus traditional open surgery has been shown. Here, we focus on minimally invasive technologies and further investigate the value of robotic versus laparoscopic renal surgery. As compared to laparoscopic technology, robotic technology has improved ergonomics and intraoperative imaging, a shorter learning curve, and better surgical maneuvering due to improved surgical dexterity [5]. Some prior studies, regardless of tumor complexity, have reported better surgical and post-operative outcomes with the use of robotic technology [1, 2, 6], while others have shown equivalent surgical and oncological outcomes [7–11].

Previous studies comparing the cost of robotic surgery to laparoscopic surgery have focused on the cost of the index surgery and the period immediately after surgery and, generally, have reported a higher cost for robotic surgery [6–8, 12–14]. This may be due to the high cost of acquisition and maintenance of robotic technology, which is reflected in the immediate perioperative cost of the surgery. There is a dearth of literature on the long-term cost implication and post-operative health care use of robotic renal surgery.

In this study, we sought to examine the temporal trends of partial nephrectomy (PN) and radical nephrectomy (RN) with adoptions of surgical approaches and examine associations with one-year healthcare expenditure and utilization. Further, we intend to compare one-year healthcare expenditures and utilization between robotic-assisted surgery (RAS) and laparoscopic surgery (Lap) in stratified samples of PN and RN cohorts.

### Patients and methods

#### Study design

An observational retrospective cohort study was conducted using the Merative^TM^ MarketScan® Research Database (MarketScan), a large de-identified employer-sponsored health insurance claims database. This is an observational study using secondary de-identified data with no possibility of identification of patient. Hence, institutional review board was not required in accordance with 45 CFR §46. The study population was kidney cancer patients aged 18 to 65 years who underwent PN or RN between July 1^st^ 2013 and December 31^st^ 2019. Codes used to identify patients and procedures are presented in eTable 1. Patients who had undergone either laparoscopic or robotic-assisted nephrectomy, and were continuously insured from 180 days pre-surgery through 365 days post-surgery were included. Continuous insurance coverage was required to longitudinally follow patients and ensure all healthcare utilizations and baseline comorbidities were captured. Exclusion criteria were inpatient cases without diagnosis-related group (DRG) code for neoplasm related kidney and ureter procedures (“656,” “657,” or “658”), bilateral nephrectomy, non-kidney cancer, secondary/metastatic cancer, severe or end-stage kidney disease, extreme total payment at index (< 1% or > 99%), or non-positive payments in the study period.

#### Outcome variables

We plotted descriptive trends of overall PN and RN, and reported percentages out of total cases performed from 2013 to 2020. Additionally, we plotted trends for robotic-assisted, laparoscopic, and open PN and RN cases and reported percentages out of total nephrectomy cases. The main outcome of the study was 1-year total healthcare expenditure. Healthcare utilization (inpatient, outpatient, and emergency room (ER)) and missed days of work due to healthcare visits were also examined. Total healthcare expenditure included payments for inpatient services, outpatient services, and prescription drug claims during the index surgery and within 365 days after discharge from patients (out of pocket) and insurance payer perspectives. All expenditures were adjusted for inflation to represent 2020 US dollars. Codes used to differentiate healthcare utilization by setting (ER, hospital, outpatient, or office-based) are shown in eTable 2. Missed days of work was estimated based on count of healthcare visit dates, assuming a one-half day of use for office-based visit, a full day of use for hospital outpatient and ER visit, and total length of stay (LOS) for inpatient admissions [15].

#### Study covariates

Patient age, sex, region, metropolitan residence status, area-level annual income status, insurance plan, year of surgery, Charlson Comorbidity Index score [16, 17] excluding kidney cancer, and baseline healthcare expenditure were considered as covariates. Insurance plans were classified into preferred provider organization (PPO), comprehensive insurance, health maintenance organization (HMO), point-of-service (POS), and other insurance plans. Baseline healthcare expenditure was the sum of payments for inpatient and outpatient services, and prescription drug payments during 180 days before surgery.

#### Statistical analysis

To reduce potential sample selection bias, inverse probability of treatment weighting (IPTW) using stabilized weights was performed separately for all comparative analyses. Logistic regression methods with all baseline characteristics included as covariates were used to estimate probability of treatment (surgical approach) for analysis cohorts. To ensure no residual differences exist between groups, baseline characteristics were compared before and after IPTW adjustment using χ^2^ test for categorical variables and Wilcoxon rank-sum test for continuous variable. Any covariate not balanced between cohorts after IPTW was added into the outcome regression models. Generalized linear models (GLM) weighted for IPTW were used to test differences of outcomes. Modified Park test was used to identify appropriate distribution family for the regression models. GLM with gamma distribution and log-link was used to compare healthcare expenditure. Binomial logistic regression was used to estimate readmission, and ER visit rates. Hospital outpatient visits, office-based provider visits, and missed days of work were estimated using zero-inflated negative-binomial regression. Sensitivity analyses were performed for estimating healthcare expenditures by excluding 1) patients who had no follow up healthcare claims, 2) patients who had multiple kidney surgeries, 3) patients with < 5 or > 95 percentile total index expenditures, and 4) patients with lower than Medicare’s expected minimum payment using Golombos et al.’s reported lower quartile of expenditures ($10,782) for radical nephrectomy among Medicare beneficiaries, after adjusting to 2020 US dollars [9]. All analyses were performed using R statistical software v4.1.2 [18]. A *p*-value of less than 0.05 was considered statistically significant.

## Results

### Nephrectomy trend analysis

**Fig. 1:**
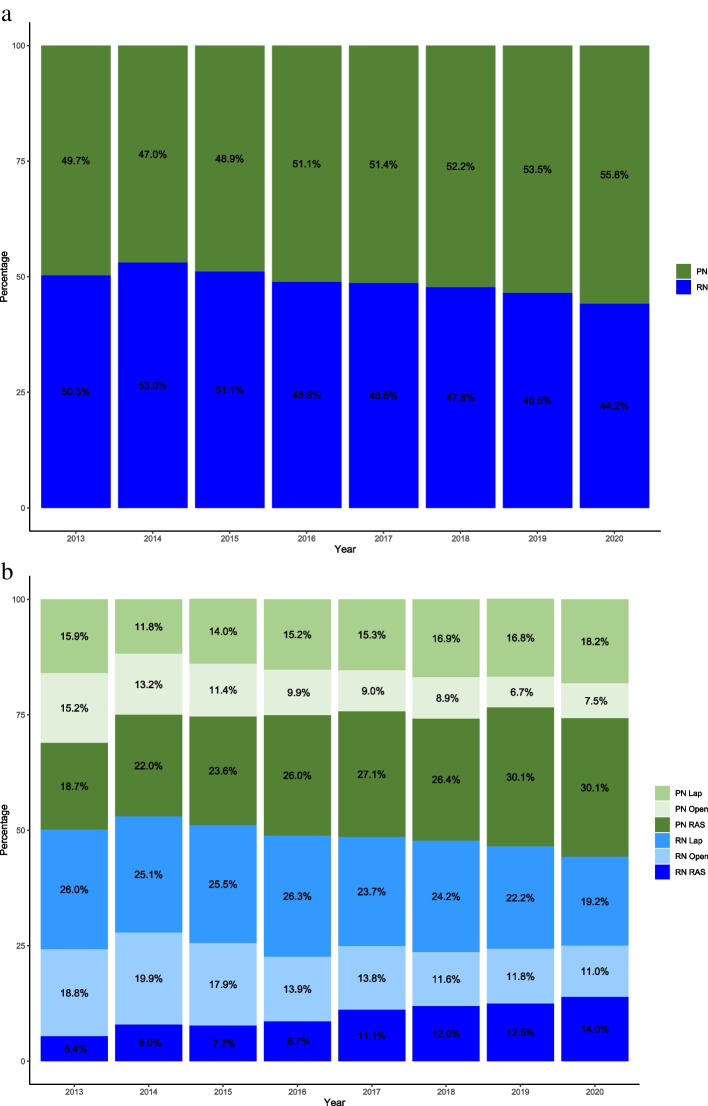
**a **Temporal trend of partial (PN) versus radical (RN) Nephrectomy. **b** Surgical approach trend of partial (PN) and radical (RN) Nephrectomy

A total of 23,532 nephrectomy cases for kidney cancer were included in the trend analysis. As shown in Fig. 1, overall adoption of PN increased over time, particularly after 2014, from 47.0% to 55.8%. The growth of PN over the years was mainly driven by increased adoption of robotic-assisted PN, which grew by 1.61-fold (18.7% in 2013 to 30.1% in 2020), while laparoscopic PN only increased by 1.14-fold from 15.9% to 18.2%, and open PN decreased from 15.2% to 7.5%. We also observed increase in robotic-assisted RN cases (5.4% in 2013 to 14.0% in 2020), along with decreases in open RN (18.8% in 2013 to 11.0% in 2020) and laparoscopic RN (26.0% in 2013 to 19.2% in 2020).

### Healthcare expenditure and utilization

**Fig. 2:**
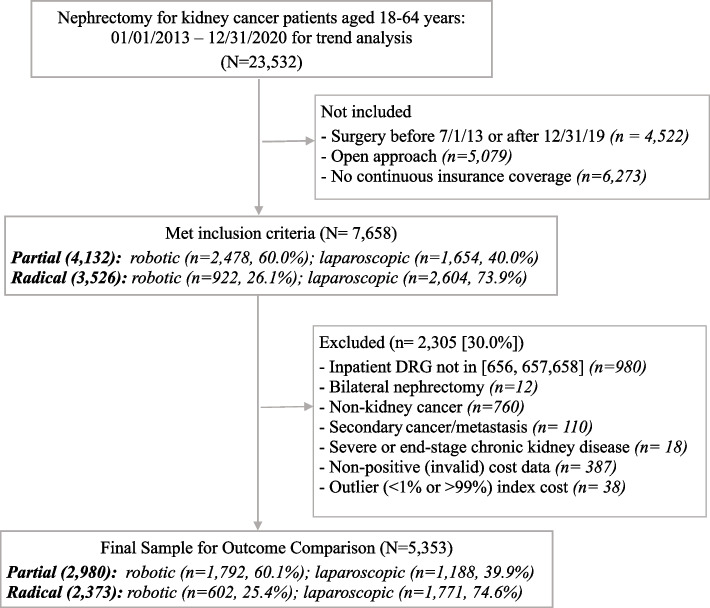
Sample selection flowchart

After applying further inclusion and exclusion criteria, the final cohort consisted of 5,353 patients who had PN (2,980 [55.7%]) or RN (2,373 [44.7%]; Fig. 2). Most of the patients were male (64.0%), aged 55 to 64 years (52.2%), and had PPO health plan (54.5%), see Table 1 for sample characteristics of PN and RN cohorts before and after IPTW. As shown in Table 2, patients who had PN had approximately one half-day shorter length of hospital stay (2.35 vs. 2.77 days, IRR = 0.85, *p* < 0.001), comparable open conversion rate (1.0% vs. 1.3%, O.R. = 0.76, *p* = 0.290), and higher total index expenditure (adjusted mean difference [AMD] = $1,117, 95% CI = $203 to $2,031, *p* = 0.017) compared to RN. In the 1-year after discharge, patients who had PN had lower readmission rate (13.9% vs. 16.8%, O.R. = 0.80, *p* = 0.003), and fewer number of hospital outpatient visits (5.0 vs. 6.5, IRR = 0.78, *p* < 0.001), resulting in lower 1-year total healthcare expenditures (AMD = -$14,111, 95% CI = -$17,756 to -$10,467, *p* < 0.001) and approximately 2 fewer days missed from work (13.2 vs. 15.3, *p* < 0.001) as compared to RN. In sensitivity analysis (eTable 3), index total expenditure was no longer significantly higher for PN as compared to RN when excluding patients with below Medicare’s expected payment. The remaining sensitivity analysis results were consistent with the main findings.

**Table 1:** Baseline characteristics of all nephrectomy patients before and after inverse probability of treatment weighting

Variable	Before IPTW				After IPTW		
	Overall, *N* = 5,353	Partial, *n* = 2,980	Radical, *n* = 2,373	*p*	Partial, *n* = 2,979	Radical, *n* = 2,374	*p*
Age, years				** < .001**			0.98
18 – 44	802 (15.0)	517 (17.3)	285 (12.0)		446 (15.0)	350 (14.8)	
45 – 54	1,760 (32.9)	974 (32.7)	786 (33.1)		979 (32.9)	782 (33.0)	
55 – 64	2,791 (52.1)	1,489 (50.0)	1,302 (54.9)		1,555 (52.2)	1,241 (52.3)	
Sex				0.300			0.94
Female	1,927 (36.0)	1,091 (36.6)	836 (35.2)		1,073 (36.0)	852 (35.9)	
Male	3,426 (64.0)	1,889 (63.4)	1,537 (64.8)		1,906 (64.0)	1,521 (64.1)	
Income, $				**0.004**			> 0.99
< 35,000	577 (10.8)	285 (9.6)	292 (12.3)		325 (10.9)	258 (10.9)	
35,000 – 39,999	1,616 (30.2)	888 (29.8)	728 (30.7)		895 (30.0)	707 (29.8)	
≥ 40,000	2,539 (47.4)	1,443 (48.4)	1,096 (46.2)		1,416 (47.5)	1,139 (48.0)	
Unknown	621 (11.6)	364 (12.2)	257 (10.8)		343 (11.5)	269 (11.3)	
Region				** < .001**			> 0.99
Northeast	900 (16.8)	571 (19.2)	329 (13.9)		500 (16.8)	402 (17.0)	
North Central	1,289 (24.1)	758 (25.4)	531 (22.4)		716 (24.0)	568 (23.9)	
West	730 (13.6)	373 (12.5)	357 (15.0)		404 (13.6)	319 (13.4)	
South	2,412 (45.1)	1,269 (42.6)	1,143 (48.2)		1,348 (45.2)	1,074 (45.3)	
Unknown	22 (0.4)	9 (0.3)	13 (0.5)		12 (0.4)	9 (0.4)	
Metro status				0.610			> 0.99
Metro	4,416 (82.5)	2,472 (83.0)	1,944 (81.9)		2,457 (82.5)	1,960 (82.6)	
Non-metro	733 (13.7)	398 (13.4)	335 (14.1)		410 (13.8)	323 (13.6)	
Unknown	204 (3.8)	110 (3.7)	94 (4.0)		112 (3.8)	90 (3.8)	
Insurance Plan				0.230			> 0.99
Comprehensive	276 (5.2)	154 (5.2)	122 (5.1)		153 (5.1)	122 (5.2)	
PPO	2,904 (54.2)	1,612 (54.1)	1,292 (54.4)		1,619 (54.4)	1,297 (54.6)	
HMO	619 (11.6)	338 (11.3)	281 (11.8)		346 (11.6)	276 (11.6)	
POS	380 (7.1)	231 (7.8)	149 (6.3)		210 (7.0)	164 (6.9)	
Others^a^	1,105 (20.6)	601 (20.2)	504 (21.2)		612 (20.6)	485 (20.4)	
Unknown	69 (1.3)	44 (1.5)	25 (1.1)		38 (1.3)	29 (1.2)	
Procedure year				0.410			0.37
2013	577 (10.8)	317 (10.6)	260 (11.0)		323 (10.8)	262 (11.1)	
2014	850 (15.9)	451 (15.1)	399 (16.8)		457 (15.4)	401 (16.9)	
2015	857 (16.0)	482 (16.2)	375 (15.8)		496 (16.6)	362 (15.2)	
2016	842 (15.7)	467 (15.7)	375 (15.8)		477 (16.0)	367 (15.5)	
2017	737 (13.8)	415 (13.9)	322 (13.6)		416 (14.0)	317 (13.4)	
2018	766 (14.3)	421 (14.1)	345 (14.5)		405 (13.6)	360 (15.2)	
2019	724 (13.5)	427 (14.3)	297 (12.5)		405 (13.6)	305 (12.8)	
CCI score				** < .001**			**0.003**
0	2,481 (46.3)	1,409 (47.3)	1,072 (45.2)		1,361 (45.7)	1,140 (48.0)	
1–2	2,220 (41.5)	1,267 (42.5)	953 (40.2)		1,283 (43.1)	919 (38.7)	
> 2	652 (12.2)	304 (10.2)	348 (14.7)		336 (11.3)	315 (13.3)	
Setting				** < .001**			0.95
Inpatient	4,634 (86.6)	2,433 (81.6)	2,201 (92.8)		2,579 (86.6)	2,053 (86.5)	
Outpatient	719 (13.4)	547 (18.4)	172 (7.2)		400 (13.4)	321 (13.5)	
Baseline expense, $				0.14			0.32
Median (IQR)	8,912.15 (4,931, 16,769)	8,735.73 (4,902, 16,312)	9,212.34 (4,951, 17,518)		8,934.26 (5,009, 16,955)	8,852.72 (4,759, 16,651)	

*Abbreviations*: *HMO* health maintenance organization, *PPO* preferred provider organization, *POS* point of service, *CCI* Charlson’s comorbidity index, *IQR* interquartile range, *SD* standard deviation

^a^Others include exclusive provider organization, consumer-drive health plan, or high deductible health plan

**Table 2:** Inverse probability of treatment weighting adjusted difference in healthcare expenditures, utilizations and estimated missed days of work between partial nephrectomy and radical nephrectomy

Outcomes	Partial, *n* = 2,979 mean (95%CI)	Radical, *n* = 2,374 mean (95%CI)	Partial vs Radical		
			OR/IRR (95%CI)	Mean difference (95%CI)	*p*
Conversion, n (%)	29 (1.0)	30 (1.3)	0.76 (0.45 to 1.27)	NA	0.290
Index LOS, d	2.35 (2.29, 2.42)	2.77 (2.69, 2.85)	0.85 (0.82 to 0.88)	-0.42 (-0.52, -0.31)	< .001
Index payment, $	32006 (31392, 32633)	30890 (30226, 31568)	NA	1,117 (203, 2031)	0.017
**1-year post-index**					
Total payment, $	21692 (20025, 23496)	35803 (32736, 39157)	NA	-14111 (-17756, -10467)	< .001
Readmission, n (%)	416 (13.9)	400 (16.8)	0.80 (0.69, 0.93)	NA	0.003
ER visit, n(%)	1,042 (35.0)	802 (33.8)	1.06 (0.94, 1.18)	NA	0.360
# Hospital OP visit	4.98 (4.78, 5.18)	6.45 (6.17, 6.73)	0.78 (0.73, 0.82)	-1.47 (-1.81, -1.12)	< .001
# Office visits	13.5 (13.1, 13.9)	14.1 (13.6, 14.5)	0.96 (0.92, 1.00)	-0.56 (-1.14, 0.02)	0.061
Missed work-days	13.2 (12.9, 13.6)	15.3 (14.8, 15.7)	NA	-2.03 (-2.63, -1.43)	< .001
**Index + 1-year post**					
Total payment, $	54342 (52147, 56630)	67612 (64560, 70808)	NA	-13270 (-17113, -9426)	< .001
Missed work-days	15.3 (14.9, 15.7)	17.7 (17.2, 18.2)	NA	-2.39 (-3.01, -1.78)	< .001

Charlson comorbidity was not balanced between cohorts after inverse probability of treatment weighting, hence added to the outcome regression models

*Abbreviations*: *LOS* length of stay, *ER* Emergency Room, *OP* Outpatient, *OR* odds ratio for conversion, readmission and ER visit, *IRR* incidence rate ratio for index LOS, and number of visits

Among 2,980 PN patients, 1,792 (60.1%) received RAS and 1,188 (39.9%) received Lap; and among 2,373 RN patients, 602 (25.4%) received RAS and 1,771 (74.6%) received Lap. Separate IPTW adjustments for PN and RN cohorts were performed to compare RAS versus Lap. Descriptive statistics of the stratified samples before and after IPTW are shown in eTable 4 (PN) and eTable 5 (RN) in the supplementary. Among the PN cohort, significantly greater proportion of RAS patients had PPO or HMO insurance plan (*p* = 0.035), were performed in 2015 or later (*p* < 0.001), and were performed as inpatient (*p* < 0.001). Among the RN cohort, a greater proportion of robotic surgeries were performed in 2017 or later (*p* < 0.001) and performed as inpatient (*p* < 0.001). After IPTW, all baseline characteristics were similar between RAS and Lap for both PN and RN cohorts.

Results for IPTW adjusted association between surgical approach and the study outcomes are presented in Table 3 (PN & RN). Among patients who had PN, RAS was associated with lower open conversion rate (0.6% vs. 1.5%, O.R. = 0.41, *p* = 0.019), shorter length of hospital stay (2.25 vs. 2.58 days, *p* < 0.001), and lower total index expenditure compared to Lap (AMD = -$1,662, 95% CI = -$2,914 to -$410, *p* = 0.009). Among patients who had RN, RAS was associated with short length of hospital stay (2.58 vs. 2.86 days, *p* = 0.003), and comparable open conversion rate (1.0% vs. 1.6%, O.R. = 0.63, *p* = 0.300) and total index expenditures ($33,105 vs. $31,524, *p* = 0.055). The one-year post-discharge total healthcare expenditures were similar between RAS and Lap for both PN ($21,400 vs. $21,874, *p* = 0.810) and RN cohorts ($38,940 vs. $43,143, *p* = 0.404). Results of the sensitivity analyses on healthcare expenditures were consistent with the main findings (eTable 6 for PN and eTable 7 for RN).

**Table 3:** Inverse probability of treatment weighting adjusted difference in healthcare expenditures, utilizations and estimated missed days of work for partial nephrectomy and radical nephrectomy

Outcomes	Lap mean (95%CI)	RAS mean (95%CI)	RAS vs Lap		
			OR/IRR (95%CI)	Mean difference (95%CI)	*p*
**Partial nephrectomy**					
** n**	1,169	1,855			
Conversion, n (%)	17 (1.5)	11 (0.6)	0.41 (0.19, 0.85)	NA	0.019
Index LOS, d	2.58 (2.47, 2.69)	2.25 (2.17, 2.33)	0.87 (0.82, 0.92)	-0.33 (-0.47, -0.20)	< 0.001
Index payment, $	32699 (31714, 33715)	31037 (30293, 31800)	NA	-1662 (-2914, -410)	0.009
**1-year post-index**					
Total payment, $	21875 (19010, 25171)	21400 (19145, 23921)	NA	-475 (-4362, 3412)	0.810
Readmission, n (%)	156 (13.3)	256 (13.8)	1.04 (0.84, 1.29)	NA	0.706
ER visit, n(%)	399 (34.2)	637 (34.3)	1.01 (0.86, 1.17)	NA	0.944
# Hospital OP visit	5.11 (4.81, 5.41)	4.70 (4.48, 4.92)	0.92 (0.85, 0.99)	-0.38 (-0.75, -0.00)	0.029
# Office visits	14.2 (13.5, 14.8)	12.9 (12.4, 13.4)	0.91 (0.86, 0.96)	-1.28 (-2.09, -0.48)	0.002
Missed work-days	13.7 (13.1, 14.3)	12.7 (12.3, 13.2)	NA	-1.00 (-1.75, -0.25)	0.008
**Index + 1-year post**					
Total payment, $	54574 (51335, 58017)	52437 (49953, 55046)	NA	-2136 (-6336, 2063)	0.316
Missed work-days	15.8 (15.2, 16.5)	14.5 (14.0, 14.9)	NA	-1.33 (-2.10, -0.57)	< 0.001
**Radical nephrectomy**					
**n**	1,775	589			
Conversion, n (%)	29 (1.6)	6 (1.0)	0.63 (0.24, 1.41)	NA	0.300
Index LOS, d	2.86 (2.76, 2.95)	2.58 (2.43, 2.74)	0.90 (0.85, 0.96)	-0.28 (-0.45, -0.10)	0.003
Index payment, $	31525 (30747, 32321)	33106 (31702, 34571)	NA	1581 (-54.9, 3217)	0.055
**1-year post-index**					
Total payment, $	43144 (38260, 48651)	38940 (31611, 47968)	NA	-4204 (-13837, 5430)	0.404
Readmission, n (%)	308 (17.3)	78 (13.2)	0.72 (0.55, 0.94)	NA	0.018
ER visit, n(%)	610 (34.4)	200 (33.9)	0.98 (0.80, 1.18)	NA	0.823
# Hospital OP visit	6.48 (6.13, 6.84)	6.80 (6.15, 7.44)	1.05 (0.94, 1.17)	0.32 (-0.42, 1.05)	0.397
# Office visits	14.2 (13.7, 14.7)	15.0 (14.1, 15.9)	1.06 (0.99, 1.13)	0.81 (-0.23, 1.84)	0.122
Missed work-days	15.5 (15.0, 16.1)	16.3 (15.3, 17.4)	NA	0.77 (-0.44, 1.99)	0.203
**Index + 1-year post**					
Total payment, $	74668 (69543, 80171)	72046 (63680, 81509)	NA	-2623 (-12979, 7734)	0.623
Missed work-days	18.2 (17.6, 18.8)	18.7 (17.7, 19.8)	NA	0.54 (-0.69, 1.77)	0.383

*Abbreviations*: *RAS* robotic-assisted Surgery, *Lap* laparoscopic surgery, *LOS* length of stay, *ER* Emergency Room, *OP* Outpatient, *OR* odds ratio for conversion, readmission and ER visit, *IRR* incidence rate ratio for index LOS, and number of visits

Despite similar 1-year total healthcare expenditures post-discharge, RAS as compared to Lap was associated with reduced healthcare utilization, with fewer hospital outpatient visits (4.72 vs. 5.12, *p* = 0.029) and fewer office-based visits (12.90 vs. 14.18, *p* = 0.002) among PN, and with reduced readmission rate (13.2% vs. 17.3%, O.R. = 0.72, *p* = 0.018) among RN. The reduced healthcare visits following PN translated to an average of one day fewer missed day of work for RAS as compared to Lap (12.71 vs. 13.71 days, *p* = 0.008).

## Discussion

Current study observed that robotic-assisted surgery has allowed surgeons to perform more PN for eligible kidney cancer patients. In a prior study, Jabaji and colleagues reported that utilization of PN increased significantly after introduction of RAS, resulting in more PN cases performed with RAS than Lap after 2012 [19]. Our study showed that utilization of PN has continued to grow from 2013 to 2020, and found that increasingly more robotic surgery practice is still driving the adoption of PN. Further, we conducted comparative analysis of healthcare expenditures, and found that PN had higher total index expenditures than RN, but one-year healthcare expenditures were significantly lower for patients who had PN as compared to RN. The difference in one-year expenditures was evident in the reduced readmission rate and outpatient healthcare visits among patients who had PN. In contrast to our finding, prior studies that compared total index expenditures reported similar total index expenditure between PN and RN patients [20, 21]. Unlike our study which assessed payer and patients’ actual payments, these studies assessed cost to the hospital/provider. We found no directly comparable study that evaluated one-year healthcare expenditures and utilization following PN and RN. A study that compared 30-day readmission and post-operative complication of PN and RN among children and young adults has reported no readmission or post-operative complication differences between PN and RN [22]. Beyond the difference in target population, Alkazemi’s findings contrast to our findings due to the shorter follow up time in their study. Evidence indicates that PN is associated with decreased risk of developing severe chronic kidney disease overtime as compared to RN, due to the preservation of kidney function with PN [23, 24]. This may explain the lower healthcare expenditure with PN in our study than with RN, as potential cost of treatment would also be averted.

The central focus of this study was to compare the one-year health expenditure of robotic assisted nephrectomy to laparoscopic nephrectomy for patients who had PN and patients who had RN. Our findings indicate that there is no significant difference in total cumulative healthcare expenditures between the two approaches at one year after discharge, but significantly fewer readmissions or outpatient visits during one year after discharge was observed with the robotic approach. When comparing index expenditures for partial nephrectomy, there was a cost saving of over $2700 with the robotic PN. As a whole, these findings are compelling, because historically the robotic platform has been derided for increased cost with similar outcome benefits compared to the laparoscopic approach. Our results suggest that not only does robotic nephrectomy, whether partial or radical, have comparable or reduced expenditures at index, but also fewer readmissions, or outpatient visits after the index surgery which may contribute to long term cost-savings.

Prior studies on radical nephrectomy index expenditure generally reported significantly higher expenditure with robotic approach than laparoscopic, in contrast to our study which found no significant difference. Using the 2009–2011 Nationwide Inpatient Sample database, Yang et al. and Gershman et al. found that robotic radical nephrectomy was associated with significantly higher total hospital cost than laparoscopic radical nephrectomy ($47,036 vs. $38,068, *p* < 0.001; $1,468 more, *p* < 0.01, respectively) [6, 25]. Using the 2010–2013 SEER-Medicare database, Golombos et al. also found that robotic radical nephrectomy was associated with higher index expenditure ($53,681 vs. $44,161, *p* < 0.01) [14]. Using the 2003–2015 Premier Healthcare database, Jeong et al. reported significantly higher 90-day direct hospital cost in their study ($19,530 vs. $16,851, *p* = 0.004), was mainly driven by higher OR and supply costs [7]. While these studies provide meaningful results using all-payer hospital cost data [6, 7, 25] and Medicare reimbursement data [14], they may not represent healthcare practice after 2015. Our study presents current status of radical nephrectomy expenditure, from commercial payers’ perspective, based on analysis of 2013–2020 MarketScan database.

Literature comparing index expenditure for partial nephrectomy between robotic and laparoscopic approaches is not as robust. Using single-center Turkish data, Haberal et al. found that the robotic approach resulted in a significantly higher procedure cost than laparoscopic approach ($17,626 vs. $4,542, *p* < 0.001) [8]. Another single-center study in China also reported higher total hospital cost with robotic partial nephrectomy as compared to laparoscopic partial nephrectomy [13]. On the contrary, U.S. based studies that compared index expenditures between robotic partial and laparoscopic partial nephrectomy using the Maryland Health Services Cost Review Commission, a state-wide hospital data [12], and using the National Inpatient Sample data [26] have found no significant difference between the two approaches. In contrast to the mentioned studies which assessed cost to the hospital/provider, our study assessed payer and patients’ actual payments.

Beyond the index period, a retrospective UK study examined the 1-year costs of open, laparoscopic, and robotic partial nephrectomy performed between 2008 and 2014, and found no significant differences between the robotic and laparoscopic approaches in terms of complication and readmission rates, as well as 1-year hospital costs [27]. A similar UK study [28] that examined 1-year and 3-year costs of open, laparoscopic, and robotic partial nephrectomy, reported that patients who had robotic partial nephrectomy had the lowest 1-year expenditure albeit no statistical significance (£779 for robotic vs. £1242 for open, *p* = 0.843; vs. £1024 for lap, no *p*-value reported). The authors reported that the main drivers for differences in 1-year expenditure were inpatient admission (£317 for robotic vs. £823 for open, *p* = 0.019; vs. £622 for lap, no *p*-value reported) and outpatient visits (£462 for robotic vs. £418 for open, *p* = 0.039; vs. £402 for lap, no *p*-value reported). The cumulative data appears to suggest that cost savings with robotic surgery, and minimally invasive approaches at large, are due to decreased complication rates [27, 29] and lower readmission rates, and emergency room presentations [27, 28]. In our prior study, we also have found that minimally invasive approaches are associated with lower readmission and hospital outpatient visits, supporting these claims [4]. In post-hoc analysis to understand impact of surgical approach on post-operative kidney function, we observed that dialysis initiation rate was trending lower for robotic versus laparoscopic partial nephrectomy especially when extending the analysis period until lost to follow up, although not significant (eTable 8 in the supplement).

With the increasing utilization of robotic technology, there is a trend in decreased cost differential between robotic and laparoscopic nephrectomy [30–32]. We hypothesize that this is due to increased surgeon proficiency with the robotic approach and attendant faster operative times. Additionally, this increased proficiency with robotic surgery appears to result in decreased healthcare utilization costs long term from decreased office and emergency department visits. This is particularly important for kidney surgery because not only does the robotic approach result in decreased healthcare utilization, but it also allows more eligible patients to undergo partial nephrectomy, preserving kidney function and reducing clinical and economic burden associated with postoperative functional decline.Strength and limitations.

Our study adds important information to the extant literature. Not only is it based on a large US commercial database that captures longitudinal payments after index surgeries, but it also describes health care service use after surgery and evaluates the change of health care cost over 1 year. We are one of the few studies that compares robotic and laparoscopic surgery for both partial and radical nephrectomy and successfully teased out the difference in index costs as well as subsequent healthcare utilization costs, including missed work days. That being said, our study also has its fair share of limitations. The retrospective design makes it difficult to account for possible unknown confounders. Given that this was an actual claims database, there is a potential risk for errors in data coding with patient identification and data extraction. Also, the results of this study may not be generalizable to older, Medicare, Medicaid, or uninsured patients. We recognize the absence of measurement and adjustment for race/ethnicity, stage of cancer, hospital characteristics (e.g., lack of robotic machines and patient volume), surgeon characteristics (e.g., skill level), and other factors that might influence the decision for surgical modality. Another caveat is our designation of days missed from work for health care visits as a proxy for time off work to seek follow-up visits, which may not truly represent lost work productivity. Lastly, we cannot ascertain whether all radical nephrectomy cases were planned, or converted from partial nephrectomy during surgery due to limitations of the data source. That being said, our results are compelling and indicate that cost savings should not be used to negate the utility of robotic nephrectomy as a viable surgical approach.

## Conclusion

During the study period, use of robotic partial nephrectomy increased tremendously, allowing more partial nephrectomy for eligible kidney cancer patients. At index period, robotic surgery was associated with reduced length of stay for both partial and radical nephrectomy, and a lower conversion rate and total expenditure for partial nephrectomy compared to the laparoscopic approach. At 1-year after discharge, while total cumulative expenditure was similar between surgical approaches, robotic approach was associated with lower readmission after radical nephrectomy, and with fewer hospital outpatient and office-based visits after partial nephrectomy compared to the laparoscopic approach.

### Supplementary Information

**Additional file 1:** **eTable** **1.** Diagnosis and Procedure codes used to identify cases. **eTable 2.** Description of place of service codes used to differentiate healthcare use. **eTable 3.** Sensitivity analyses on healthcare expenditure outcome comparisons between partial and radical nephrectomy. **eTable 4.**  Baseline characteristics of partial nephrectomy patients before and after inverse probability of treatment weighting. **eTable 5.** Baseline characteristics of radical nephrectomy patients before and after inverse probability of treatment weighting. **eTable 6.** Sensitivity analyses on healthcare expenditure outcome comparisons between robotic-assisted and laparoscopic surgery among patients who had partial nephrectomy. **eTable 7.** Sensitivity analyses on healthcare expenditure outcome comparisons between robotic-assisted and laparoscopic surgery among patients who had radical nephrectomy. **eTable 8.** Post-surgical kidney function outcomes.

## Data Availability

Data for the study is publicly available at https://www.merative.com/real-world-evidence.
