# Supplementary material for: One-year healthcare costs after robotic-assisted and laparoscopic partial and radical nephrectomy: a cohort study
Source: BMC Health Serv Res. 2023 Oct 14;23:1099. doi: 10.1186/s12913-023-10111-8 (PMC10576279; doi:10.1186/s12913-023-10111-8)
Supplement: Supplementary file 1 — Additional file 1: eTable 1. Diagnosis and Procedure codes used to identify cases. eTable 2. Description of place of service codes used to differentiate healthcare use. eTable 3. Sensitivity analyses on healthcare expenditure outcome comparisons between partial and radical nephrectomy. eTable 4. Baseline characteristics of partial nephrectomy patients before and after inverse probability of treatment weighting. eTable 5. Baseline characteristics of radical nephrectomy patients before and after inverse probability of treatment weighting. eTable 6. Sensitivity analyses on healthcare expenditure outcome comparisons between robotic-assisted and laparoscopic surgery among patients who had partial nephrectomy. eTable 7. Sensitivity analyses on healthcare expenditure outcome comparisons between robotic-assisted and laparoscopic surgery among patients who had radical nephrectomy. eTable 8. Post-surgical kidney function outcomes. [file 12913_2023_10111_MOESM1_ESM.docx]

Supplementary Materials

eTable 1. Diagnosis and Procedure codes used to identify cases.

eTable 2. Description of place of service codes used to differentiate healthcare use.

eTable 3. Sensitivity analyses on healthcare expenditure outcome comparisons between partial and radical nephrectomy

eTable 4. Baseline characteristics of partial nephrectomy patients before and after inverse probability of treatment weighting

eTable 5. Baseline characteristics of radical nephrectomy patients before and after inverse probability of treatment weighting

eTable 6. Sensitivity analyses on healthcare expenditure outcome comparisons between robotic-assisted and laparoscopic surgery among patients who had partial nephrectomy.

eTable 7. Sensitivity analyses on healthcare expenditure outcome comparisons between robotic-assisted and laparoscopic surgery among patients who had radical nephrectomy.

eTable 8. Post-surgical kidney function outcomes

eTable 1. Diagnosis and Procedure codes used to identify cases

| Variables | Diagnosis and procedure Codes by Code Type | | |
| --- | --- | --- | --- |
|  | ICD-9 | ICD-10 | CPT/HCPCS |
| Type of nephrectomy |  |  |  |
| Partial | 55.4 | 0TB04ZZ, 0TB14ZZ, 0TB00ZZ, 0TB10ZZ | 50543, 50240 |
| Radical | 55.51, 55.52 | 0TT04ZZ, 0TT14ZZ, 0TT00ZZ, 0TT10ZZ | 50545, 50546, 50230 |
| Bilateral | 55.54 | 0TT20ZZ, 0TT24ZZ |  |
| Surgical approach |  |  |  |
| RAS^1^ | **17.41, 17.42, 17.43, 17.44, 17.45, 17.49** | **8E0W0CZ, 8E0W3CZ, 8E0W4CZ,  8E0W7CZ, 8E0W8CZ, 8E0WXCZ** | **S2900** |
| Lap | **54.21, 54.51** | 0TB04ZZ, 0TB14ZZ, 0TT04ZZ, 0TT14ZZ | 50543, 50545, 50546 |
| Diagnoses |  |  |  |
| Malignant kidney tumor | 189.0 | C64.1, C64.2, C64.9 | - |
| Severe CKD^2^ | 585.4, 585.5, 585.6 | N18.4, N18.5, N18.6 | - |

^1^ Codes in **bold** were used as modifier codes for robotic-assisted (RAS) or laparoscopic surgery (Lap) with a presence of nephrectomy code.
^2^ Severe chronic kidney disease (CKD) includes stage IV, stage V, and end-stage renal disease.
**Note**: Presence of RAS modifier code supersedes in defining surgical approach.

eTable 2. Description of place of service codes used to differentiate healthcare use

| Type of Healthcare visit | Place of service code (STDPLAC) | Label |
| --- | --- | --- |
| Emergency/Urgent care visit^1^ | 20  23 | Urgent care facility  Emergency room – hospital |
| Hospital Outpatient visit | 19  22  24  95 | Outpatient Hospital-Off Campus  Outpatient Hospital-On Campus  Ambulatory Surgical Center  Outpatient (NEC) |
| Office-based provider visit | 11 | Office |

^1^ In addition, healthcare encounters with emergency room service category code (SVCSCAT) ending with “20” were considered emergency visits; For example: “12220” - Facility OP ER

eTable 3. Sensitivity analyses on healthcare expenditure outcome comparisons between partial and radical nephrectomy

| Outcomes | Partial nephrectomy | Radical nephrectomy |  | Adjusted difference (Lap as reference) | |
| --- | --- | --- | --- | --- | --- |
|  | mean (95%CI) | mean (95%CI) |  | Mean (95%CI) | *P* |
| *Excluding 5% extreme index expenditure ^1^* |  |  |  |  |  |
| Index, $ | 31236 (30740, 31740) | 30294 (29750, 30848) |  | 942 (200, 1685) | < .001 |
| 1-year post, $ | 21268 (19596, 23083) | 34319 (31277, 37656) |  | -13051 (-16681, -9421) | < .001 |
| Index + 1-year post, $ | 53046 (50902, 55281) | 65243 (62263, 68366) |  | -12197 (-15951, -8443) | < .001 |
| *Excluding below Medicare payment ^2^* |  |  |  |  |  |
| Index, $ | 34335 (33733, 34948) | 33691 (33025, 34372) |  | 644 (-263, 1550) | 0.164 |
| 1-year post, $ | 21411 (19673, 23302) | 35800 (32532, 39395) |  | -14389 (-18265, -10513) | < .001 |
| Index + 1-year post, $ | 56395 (54074, 58817) | 70324 (67059, 73747) |  | -13928 (-18026, -9830) | < .001 |
| *Excluding no claim for follow up care ^3^* |  |  |  |  |  |
| Index, $ | 32021 (31406, 32648) | 30897 (30233, 31576) |  | 1124 (209, 2039) | 0.016 |
| 1-year post, $ | 21719 (20051, 23526) | 35854 (32783, 39213) |  | -14135 (-17785, -10486) | < .001 |
| Index + 1-year post, $ | 54384 (52187, 56674) | 67668 (64611, 70868) |  | -13283 (-17132, -9435) | < .001 |
| *Excluding multiple surgery cases ^4^* |  |  |  |  |  |
| Index, $ | 32296 (31683, 32921) | 31280 (30616, 31958) |  | 1016 (103, 1929) | 0.029 |
| 1-year post, $ | 20301 (18656, 22091) | 35005 (31848, 38476) |  | -14704 (-18432, -10977) | < .001 |
| Index + 1-year post, $ | 53224 (51014, 55529) | 67175 (64063, 70438) |  | -13951 (-17856, -10047) | < .001 |

^1^ N=205 patients were excluded; ^2^ N = 520 patients were excluded; ^3^ N = 6 patients were excluded; ^4^ N = 221 patients were excluded

eTable 4. Baseline characteristics of partial nephrectomy patients before and after inverse probability of treatment weighting

| Variable | Before | | | |  | After | | |
| --- | --- | --- | --- | --- | --- | --- | --- | --- |
|  | All (n = 2,980) | Lap (n= 1,188) | RAS (n= 1,792) | *p* |  | Lap (n= 1,168) | RAS (n= 1,855) | *p* |
| Age, years |  |  |  | 0.110 |  |  |  | 0.910 |
| 18 – 44 | 517 (17.3) | 225 (18.9) | 292 (16.3) |  |  | 204 (17.4) | 336 (18.1) |  |
| 45 – 54 | 974 (32.7) | 369 (31.1) | 605 (33.8) |  |  | 382 (32.7) | 608 (32.8) |  |
| 55 – 64 | 1,489 (50.0) | 594 (50.0) | 895 (49.9) |  |  | 583 (49.9) | 911 (49.1) |  |
| Sex |  |  |  | 0.430 |  |  |  | 0.530 |
| Female | 1,091 (36.6) | 445 (37.5) | 646 (36.0) |  |  | 434 (37.1) | 664 (35.8) |  |
| Male | 1,889 (63.4) | 743 (62.5) | 1,146 (64.0) |  |  | 734 (62.9) | 1,191 (64.2) |  |
| Income, $ |  |  |  | 0.270 |  |  |  | 0.980 |
| < 35,000 | 285 (9.6) | 112 (9.4) | 173 (9.7) |  |  | 115 (9.8) | 176 (9.5) |  |
| 35,000 – 39,999 | 888 (29.8) | 375 (31.6) | 513 (28.6) |  |  | 342 (29.3) | 532 (28.7) |  |
| ≥ 40,000 | 1,443 (48.4) | 560 (47.1) | 883 (49.3) |  |  | 575 (49.2) | 927 (50.0) |  |
| Unknown | 364 (12.2) | 141 (11.9) | 223 (12.4) |  |  | 136 (11.6) | 219 (11.8) |  |
| Region |  |  |  | **<.001** |  |  |  | 0.960 |
| Northeast | 571 (19.2) | 229 (19.3) | 342 (19.1) |  |  | 233 (19.9) | 355 (19.2) |  |
| North Central | 758 (25.4) | 278 (23.4) | 480 (26.8) |  |  | 275 (23.5) | 452 (24.4) |  |
| West | 373 (12.5) | 148 (12.5) | 225 (12.6) |  |  | 138 (11.8) | 212 (11.4) |  |
| South | 1,269 (42.6) | 529 (44.5) | 740 (41.3) |  |  | 519 (44.4) | 832 (44.8) |  |
| Unknown | 9 (0.3) | 4 (0.3) | 5 (0.3) |  |  | 4 (0.3) | 4 (0.2) |  |
| Metro status |  |  |  | 0.550 |  |  |  | 0.920 |
| Metro | 2,472 (83.0) | 985 (82.9) | 1,487 (83.0) |  |  | 979 (83.8) | 1,556 (83.9) |  |
| Non-metro | 398 (13.4) | 164 (13.8) | 234 (13.1) |  |  | 151 (12.9) | 244 (13.2) |  |
| Unknown | 110 (3.7) | 39 (3.3) | 71 (4.0) |  |  | 38 (3.2) | 55 (3.0) |  |
| Insurance Plan |  |  |  | **0.035** |  |  |  | >0.99 |
| Comprehensive | 154 (5.2) | 46 (3.9) | 108 (6.0) |  |  | 53 (4.6) | 87 (4.7) |  |
| PPO | 1,612 (54.1) | 638 (53.7) | 974 (54.4) |  |  | 630 (54.0) | 985 (53.1) |  |
| HMO | 338 (11.3) | 125 (10.5) | 213 (11.9) |  |  | 131 (11.2) | 203 (10.9) |  |
| POS | 1,612 (54.1) | 638 (53.7) | 974 (54.4) |  |  | 92 (7.9) | 154 (8.3) |  |
| Others ^a^ | 601 (20.2) | 258 (21.7) | 343 (19.1) |  |  | 244 (20.9) | 403 (21.7) |  |
| Unknown | 44 (1.5) | 19 (1.6) | 25 (1.4) |  |  | 17 (1.4) | 24 (1.3) |  |

eTable 4. Continued

| Variable | Before | | | |  | After | | |
| --- | --- | --- | --- | --- | --- | --- | --- | --- |
|  | All (n =2,980) | Lap (n= 1,188) | RAS (n= 1,792) | *p* |  | Lap (n= 1,168) | RAS (n= 1,855) | *p* |
| Procedure year |  |  |  | **<.001** |  |  |  | 0.320 |
| 2013 | 317 (10.6) | 162 (13.6) | 155 (8.6) |  |  | 137 (11.7) | 222 (12.0) |  |
| 2014 | 451 (15.1) | 206 (17.3) | 245 (13.7) |  |  | 186 (15.9) | 283 (15.2) |  |
| 2015 | 482 (16.2) | 179 (15.1) | 303 (16.9) |  |  | 181 (15.5) | 314 (16.9) |  |
| 2016 | 467 (15.7) | 165 (13.9) | 302 (16.9) |  |  | 161 (13.8) | 313 (16.9) |  |
| 2017 | 415 (13.9) | 155 (13.0) | 260 (14.5) |  |  | 182 (15.5) | 233 (12.5) |  |
| 2018 | 421 (14.1) | 164 (13.8) | 257 (14.3) |  |  | 166 (14.3) | 249 (13.4) |  |
| 2019 | 427 (14.3) | 157 (13.2) | 270 (15.1) |  |  | 155 (13.2) | 242 (13.1) |  |
| CCI score |  |  |  | 0.330 |  |  |  | 0.340 |
| 0 | 1,409 (47.3) | 581 (48.9) | 828 (46.2) |  |  | 561 (48.1) | 873 (47.1) |  |
| 1-2 | 1,267 (42.5) | 487 (41.0) | 780 (43.5) |  |  | 481 (41.2) | 812 (43.8) |  |
| >2 | 304 (10.2) | 120 (10.1) | 184 (10.3) |  |  | 125 (10.7) | 170 (9.1) |  |
| Setting |  |  |  | **<.001** |  |  |  | 0.230 |
| Inpatient | 2,433 (81.6) | 728 (61.3) | 1,705 (95.1) |  |  | 948 (81.2) | 1,459 (78.7) |  |
| Outpatient | 547 (18.4) | 460 (38.7) | 87 (4.9) |  |  | 219 (18.8) | 396 (21.3) |  |
| Baseline expense, $ |  |  |  | 0.086 |  |  |  | 0.130 |
| Median (IQR) | 8,736  (4,902, 16,312) | 9,004  (5,016, 17,239) | 8,519  (4,864, 15,389) |  |  | 8,972  (4,840, 17,105) | 8,222  (4,790, 15,162) |  |
| Mean (SD) | 14,686  (20,971) | 15,159 (18,765.33) | 14,372  (22,313) |  |  | 15,052  (18,452) | 14,562  (23,481) |  |

**Abbreviations**: RAS, robotic-assisted surgery; Lap, laparoscopic surgery; HMO, health maintenance organization; PPO, preferred provider organization, POS, point of service; CCI, Charlson’s comorbidity index; IQR, interquartile range; SD, standard deviation

^a^Others include exclusive provider organization, consumer-drive health plan, or high deductible health plan

eTable 5. Baseline characteristics of radical nephrectomy patients before and after inverse probability of treatment weighting

| Variable | Before | | | |  | After | | |
| --- | --- | --- | --- | --- | --- | --- | --- | --- |
|  | All (n = 2,373) | Lap (n= 1,771) | RAS (n= 602) | *p* |  | Lap (n= 1,775) | RAS (n= 589) | *p* |
| Age, years |  |  |  | 0.870 |  |  |  | 0.930 |
| 18 – 44 | 285 (12.0) | 210 (11.9) | 75 (12.5) |  |  | 213 (12.0) | 71 (12.1) |  |
| 45 – 54 | 786 (33.1) | 591 (33.4) | 195 (32.4) |  |  | 588 (33.1) | 200 (34.0) |  |
| 55 – 64 | 1,302 (54.9) | 970 (54.8) | 332 (55.1) |  |  | 974 (54.9) | 318 (53.9) |  |
| Sex |  |  |  | 0.092 |  |  |  | 0.860 |
| Female | 836 (35.2) | 641 (36.2) | 195 (32.4) |  |  | 623 (35.1) | 204 (34.7) |  |
| Male | 1,537 (64.8) | 1,130 (63.8) | 407 (67.6) |  |  | 1,152 (64.9) | 385 (65.3) |  |
| Income, $ |  |  |  | 0.140 |  |  |  | 0.990 |
| < 35,000 | 292 (12.3) | 232 (13.1) | 60 (10.0) |  |  | 218 (12.3) | 70 (11.8) |  |
| 35,000 – 39,999 | 728 (30.7) | 540 (30.5) | 188 (31.2) |  |  | 544 (30.7) | 182 (30.8) |  |
| ≥ 40,000 | 1,096 (46.2) | 802 (45.3) | 294 (48.8) |  |  | 821 (46.3) | 277 (47.0) |  |
| Unknown | 257 (10.8) | 197 (11.1) | 60 (10.0) |  |  | 191 (10.8) | 61 (10.4) |  |
| Region |  |  |  | **<.001** |  |  |  | 0.970 |
| Northeast | 329 (13.9) | 253 (14.3) | 76 (12.6) |  |  | 243 (13.7) | 76 (13.0) |  |
| North Central | 531 (22.4) | 337 (19.0) | 194 (32.2) |  |  | 400 (22.5) | 134 (22.7) |  |
| West | 357 (15.0) | 271 (15.3) | 86 (14.3) |  |  | 268 (15.1) | 92 (15.6) |  |
| South | 1,143 (48.2) | 899 (50.8) | 244 (40.5) |  |  | 853 (48.1) | 285 (48.4) |  |
| Unknown | 13 (0.5) | 11 (0.6) | 2 (0.3) |  |  | 10 (0.5) | 2 (0.3) |  |
| Metro status |  |  |  | 0.440 |  |  |  | 0.980 |
| Metro | 1,944 (81.9) | 1,453 (82.0) | 491 (81.6) |  |  | 1,453 (81.8) | 481 (81.6) |  |
| Non-metro | 335 (14.1) | 253 (14.3) | 82 (13.6) |  |  | 251 (14.2) | 83 (14.1) |  |
| Unknown | 94 (4.0) | 65 (3.7) | 29 (4.8) |  |  | 71 (4.0) | 25 (4.2) |  |
| Insurance Plan |  |  |  | 0.540 |  |  |  | >0.99 |
| Comprehensive | 122 (5.1) | 84 (4.7) | 38 (6.3) |  |  | 91 (5.1) | 29 (5.0) |  |
| PPO | 1,292 (54.4) | 977 (55.2) | 315 (52.3) |  |  | 968 (54.5) | 323 (54.8) |  |
| HMO | 281 (11.8) | 204 (11.5) | 77 (12.8) |  |  | 212 (11.9) | 69 (11.7) |  |
| POS | 149 (6.3) | 112 (6.3) | 37 (6.1) |  |  | 111 (6.2) | 38 (6.4) |  |
| Others ^a^ | 504 (21.2) | 377 (21.3) | 127 (21.1) |  |  | 375 (21.1) | 124 (21.1) |  |
| Unknown | 25 (1.1) | 17 (1.0) | 8 (1.3) |  |  | 19 (1.1) | 6 (1.0) |  |

eTable 5. Continued

| Variable | Before | | | |  | After | | |
| --- | --- | --- | --- | --- | --- | --- | --- | --- |
|  | All (n = 2,373) | Lap (n= 1,771) | RAS (n= 602) | *p* |  | Lap (n= 1,775) | RAS (n= 589) | *p* |
| Procedure year |  |  |  | **<.001** |  |  |  | 0.210 |
| 2013 | 260 (11.0) | 226 (12.8) | 34 (5.6) |  |  | 197 (11.1) | 57 (9.7) |  |
| 2014 | 399 (16.8) | 329 (18.6) | 70 (11.6) |  |  | 297 (16.7) | 96 (16.3) |  |
| 2015 | 375 (15.8) | 300 (16.9) | 75 (12.5) |  |  | 285 (16.1) | 81 (13.7) |  |
| 2016 | 375 (15.8) | 282 (15.9) | 93 (15.4) |  |  | 278 (15.7) | 90 (15.2) |  |
| 2017 | 322 (13.6) | 205 (11.6) | 117 (19.4) |  |  | 219 (12.3) | 102 (17.3) |  |
| 2018 | 345 (14.5) | 226 (12.8) | 119 (19.8) |  |  | 258 (14.5) | 88 (15.0) |  |
| 2019 | 297 (12.5) | 203 (11.5) | 94 (15.6) |  |  | 241 (13.6) | 76 (12.8) |  |
| CCI score |  |  |  | 0.230 |  |  |  | 0.450 |
| 0 | 1,072 (45.2) | 805 (45.5) | 267 (44.4) |  |  | 795 (44.8) | 274 (46.5) |  |
| 1-2 | 953 (40.2) | 719 (40.6) | 234 (38.9) |  |  | 723 (40.7) | 222 (37.6) |  |
| >2 | 348 (14.7) | 247 (13.9) | 101 (16.8) |  |  | 257 (14.5) | 93 (15.8) |  |
| Setting |  |  |  | **<.001** |  |  |  | 0.650 |
| Inpatient | 2,201 (92.8) | 1,609 (90.9) | 592 (98.3) |  |  | 1,647 (92.8) | 552 (93.7) |  |
| Outpatient | 172 (7.2) | 162 (9.1) | 10 (1.7) |  |  | 128 (7.2) | 37 (6.3) |  |
| Baseline expense, $ |  |  |  | 0.260 |  |  |  | 0.690 |
| Median (IQR) | 9,212 (4,951, 17,518) | 9,166  (4,904, 17,121) | 9,305  (5,055, 18,652) |  |  | 9,253  (4,960, 17,393) | 9,228  (5,063, 17,717) |  |
| Mean (SD) | 18,371  (39,883) | 17,584  (34,477) | 20,687  (52,632) |  |  | 18,235  (36,218) | 18,125  (40,469) |  |

**Abbreviations**: RAS, robotic-assisted surgery; Lap, laparoscopic surgery; HMO, health maintenance organization; PPO, preferred provider organization, POS, point of service; CCI, Charlson’s comorbidity index; IQR, interquartile range; SD, standard deviation

^a^Others include exclusive provider organization, consumer-drive health plan, or high deductible health plan

eTable 6. Sensitivity analyses on healthcare expenditure outcome comparisons between robotic-assisted and laparoscopic surgery among patients who had partial nephrectomy.

| Outcomes | RAS | Lap |  | Adjusted difference (Lap as reference) | |
| --- | --- | --- | --- | --- | --- |
|  | mean (95%CI) | mean (95%CI) |  | Mean (95%CI) | *P* |
| *Excluding 5% extreme index expenditure ^1^* |  |  |  |  |  |
| Index, $ | 30303 (29660, 30959) | 32311 (31449, 33198) |  | -2009 (-3098, -920) | < .001 |
| 1-year post, $ | 20939 (18664, 23492) | 21546 (18635, 24913) |  | -607 (-4555, 3341) | 0.762 |
| Index + 1-year post, $ | 51242 (48773, 53835) | 53858 (50605, 57320) |  | -2616 (-6818, 1586) | 0.219 |
| *Excluding below Medicare payment ^2^* |  |  |  |  |  |
| Index, $ | 33444 (32712, 34192) | 35796 (34803, 36817) |  | -2352 (-3602, -1103) | < .001 |
| 1-year post, $ | 21293 (18915, 23971) | 20958 (18028, 24365) |  | 335 (-3705, 4375) | 0.871 |
| Index + 1-year post, $ | 54737 (52116, 57490) | 56754 (53321, 60408) |  | -2017 (-6462, 2427) | 0.371 |
| *Excluding no claim for follow up care ^3^* |  |  |  |  |  |
| Index, $ | 31055 (30311, 31819) | 32699 (31714, 33714) |  | -1644 (-2896, -392) | 0.010 |
| 1-year post, $ | 21431 (19172, 23955) | 21875 (19012, 25169) |  | -444 (-4331, 3443) | 0.822 |
| Index + 1-year post, $ | 52486 (49998, 55098) | 54574 (51336, 58016) |  | -2088 (-6288, 2112) | 0.328 |
| *Excluding multiple surgery cases ^4^* |  |  |  |  |  |
| Index, $ | 31435 (30690, 32198) | 33293 (32303, 34313) |  | -1858 (-3114, -601) | 0 .004 |
| 1-year post, $ | 20222 (17950, 22782) | 20543 (17683, 23865) |  | -321 (-4231, 3590) | 0.872 |
| Index + 1-year post, $ | 51657 (49156, 54285) | 53835 (50577, 57303) |  | -2179 (-6406, 2049) | 0.310 |

^1^ N = 105 patients were excluded; ^2^ N = 318 patients were excluded; ^3^ N = 3 patients were excluded; ^4^ N = 150 patients were excluded

**Abbreviations**: RAS, Robotic-assisted Surgery; Lap, Laparoscopic surgery

eTable 7. Sensitivity analyses on healthcare expenditure outcome comparisons between robotic-assisted and laparoscopic surgery among patients who had radical nephrectomy.

| Outcomes | RAS | Lap |  | Adjusted difference (Lap as reference) | |
| --- | --- | --- | --- | --- | --- |
|  | mean (95%CI) | mean (95%CI) |  | Mean (95%CI) | *P* |
| *Excluding 5% extreme index expenditure ^1^* |  |  |  |  |  |
| Index, $ | 31308 (30238, 32416) | 30650 (30040, 31271) |  | 659 (-592, 1909) | 0.299 |
| 1-year post, $ | 34443 (28037, 42313) | 41070 (36469, 46252) |  | -6627 (-15233, 1978) | 0.147 |
| Index + 1-year post, $ | 65751 (58372, 74063) | 71720 (66956, 76824) |  | -5969 (-15219, 3282) | 0.215 |
| *Excluding below Medicare payment ^2^* |  |  |  |  |  |
| Index, $ | 34896 (33516, 36334) | 33682 (32902, 34481) |  | 1214 (-401, 2828) | 0.137 |
| 1-year post, $ | 39023 (31338, 48593) | 43297 (38122, 49176) |  | -4274 (-14454, 5906) | 0.422 |
| Index + 1-year post, $ | 73919 (65132, 83891) | 76980 (71528, 82847) |  | -3061 (-13991, 7870) | 0.587 |
| *Excluding no claim for follow up care ^3^* |  |  |  |  |  |
| Index, $ | 33106 (31702, 34572) | 31533 (30755, 32330) |  | 1573 (-63.8, 3209) | 0.056 |
| 1-year post, $ | 38940 (31614, 47963) | 43209 (38317, 48727) |  | -4269 (-13904, 5365) | 0.397 |
| Index + 1-year post, $ | 72046 (63682, 81508) | 74742 (69609, 80254) |  | -2696 (-13056, 7663) | 0.613 |
| *Excluding multiple surgery cases ^4^* |  |  |  |  |  |
| Index, $ | 33164 (31768, 34621) | 31720 (30935, 32524) |  | 1444 (-188, 3077) | 0.080 |
| 1-year post, $ | 38872 (31385, 48147) | 42130 (37194, 47721) |  | -3258 (-13093, 6578) | 0.524 |
| Index + 1-year post, $ | 72036 (63544, 81663) | 73850 (68647, 79447) |  | -1814 (-12337, 8710) | 0.737 |

^1^ N = 100 patients were excluded; ^2^ N = 202 patients were excluded; ^3^ N = 3 patients were excluded; ^4^ N = 71 patients were excluded

**Abbreviations**: RAS, Robotic-assisted Surgery; Lap, Laparoscopic surgery

eTable 8. Post-surgical kidney function outcomes

| Outcomes | Partial Nephrectomy | | | |  | Radical Nephrectomy | | | |
| --- | --- | --- | --- | --- | --- | --- | --- | --- | --- |
|  | RAS n (%) | Lap n (%) | OR/HR (95%CI) | *p* |  | RAS n (%) | Lap n (%) | OR/HR (95%CI) | *p* |
| Post-discharge 1-year |  |  |  |  |  |  |  |  |  |
| Dialysis | 6 (0.3) | 3 (0.2) | 1.36 (0.33, 7.30) | 0.685 |  | 5 (0.9) | 18 (1.0) | 0.86 (0.29, 2.15) | 0.766 |
| Severe stage CKD | 14 (0.7) | 11 (1.0) | 0.75 (0.34, 1.67) | 0.467 |  | 7 (1.2) | 37 (2.1) | 0.56 (0.23, 1.19) | 0.162 |
| Any stage CKD | 91 (4.9) | 58 (5.0) | 0.98 (0.70, 1.38) | 0.919 |  | 92 (15.6) | 301 (17.0) | 0.90 (0.70, 1.16) | 0.430 |
| Until lost-to-follow up |  |  |  |  |  |  |  |  |  |
| Dialysis | 17 (0.9) | 21 (1.8) | 0.54 (0.27, 1.06) | 0.076 |  | 11 (1.9) | 34 (1.9) | 1.04 (0.54, 2.01) | 0.899 |
| Severe stage CKD | 28 (1.5) | 22 (1.9) | 0.82 (0.46, 1.47) | 0.511 |  | 18 (3.1) | 78 (4.4) | 0.73 (0.43, 1.24) | 0.245 |
| Any stage CKD | 177 (9.5) | 127 (10.9) | 0.90 (0.68 1.18) | 0.441 |  | 165 (28.0) | 493 (27.8) | 1.04 (0.86, 1.26) | 0.683 |

**Abbreviations**: RAS, robotic-assisted Surgery; Lap, laparoscopic surgery; LOS, length of stay, ER, Emergency Room, OP, Outpatient; OR, odds ratio for post-discharge 1-year outcomes; HR, hazard ratio for until lost-to-follow up outcomes
